# Could We Have Improved on ADAURA? Refining Global Research Practices to Reduce Cancer Care Disparities

**DOI:** 10.1200/GO.23.00344

**Published:** 2023-11-22

**Authors:** Howard (Jack) West, Amol Akhade, Bishal Gyawali

**Affiliations:** ^1^ Department of Medical Oncology, City of Hope Comprehensive Cancer Center, Duarte, CA; ^2^ AccessHope, Los Angeles, CA; ^3^ Department of Medical Oncology, Nair Hospital and Topiwala National Medical College Mumbai, Mumbai, India; ^4^ Suyog Cancer Clinics, Mumbai, India; ^5^ Departments of Oncology and Public Health Sciences, Queen's University, Kingston, Canada; ^6^ Division of Cancer Care and Epidemiology, Queen's University, Kingston, Canada

## Abstract

ADAURA was a positive trial, but could changes have improved it and reduced global care disparities?

## Introduction

The ADAURA trial of adjuvant osimertinib versus placebo for up to 3 years in patients with resected stage IB-IIIA epidermal growth factor receptor (*EGFR*) mutation–positive (*EGFRm+*) non–small-cell lung cancer (NSCLC) was initiated in 2015, before osimertinib was approved and routinely used in advanced *EGFRm+* NSCLC, with disease-free survival (DFS) as the primary end point and without crossover.^1^ An interim analysis in 2020 led to the trial being unblinded and preliminary results of a highly significant improvement in DFS being presented at ASCO 2020.^1^ The study follow-up was still ongoing, awaiting overall survival (OS) outcomes.

## DFS as a Suboptimal End Point

At that time, we argued that DFS was a suboptimal end point because OS represents the decisive end point in a curative setting^2^ and because preceding trials of adjuvant EGFR tyrosine kinase inhibitors (TKIs) had repeatedly demonstrated a significant improvement in DFS without improving OS.^3^ Nevertheless, the US Food and Drug Administration (FDA) considered DFS as an acceptable end point and approved adjuvant osimertinib for the ADAURA-eligible population. This led to its broad adoption on the basis of the presumption that the DFS benefit, especially with a hazard ratio (HR) of 0.17, would be highly likely to herald a significant improvement in OS.^1^ However, we argued that a DFS benefit in the midst of ongoing treatment does not necessarily connote sustained benefit beyond the time of active treatment,^2^ and in fact, the pattern of the DFS curves with longer follow-up revealed the DFS benefit eroding promptly after the treatment interval ends.^4^

By 2020, osimertinib had already been shown to be significantly superior to first-generation EGFR TKIs not only for progression-free survival (PFS)^5^ but also for OS^6^ as first-line treatment for advanced *EGFRm+* NSCLC and was the first-line standard of care in this setting. Accordingly, the most relevant contemporary question from ADAURA became whether proactive administration of adjuvant osimertinib improves OS versus osimertinib initiated at relapse as needed, regardless of whether the FDA accepted a lower standard. ADAURA was amended in April, 2020 to permit (but not mandate) crossover of patients with relapsed disease to osimertinib on request.

## OS Benefit Demonstrated With Caveats

Updated results from ADAURA highlight a significant improvement in OS with adjuvant osimertinib (HR, 0.49, 5-year OS for trial population 88% *v* 78%, *P* < .0001).^7^ These results are practice affirming for oncologists who had adopted adjuvant osimertinib on the basis of the DFS benefit and reassuring to those holding out for an OS benefit. However, only 79 of 205 (38.5%) patients on the control arm whose disease relapsed received osimertinib. Although there is always a risk of attrition from patients experiencing clinical decline that precludes them receiving treatment later, the high proportion of patients on the control arm receiving an inferior, earlier generation EGFR TKI (56% of patients with relapse) underscores that the low rate of osimertinib administration was *not due to attrition*. Instead, the pattern of postprotocol treatment must be interpreted as reflecting challenges in accessing osimertinib by crossover at physician request.

The inferior postprotocol therapy did not raise regulatory flags and should not be presumed to undermine the observed OS benefit, but the comparison of the experimental arm is now anchored by the lowest global standard of care that can be justified. Unfortunately, the low rate of crossover to osimertinib illustrated in the patterns of postprotocol treatment in ADAURA confounds osimertinib timing (as adjuvant therapy for all *v* treatment as needed for a subgroup at relapse) versus overall access to osimertinib, thereby compromising the interpretability of the study relative to current practice.

## Can We Do Better? Maximizing Alignment Among Different Stakeholders

Sponsor companies, health care professionals, and patients share many common goals when considering clinical trials, but we should acknowledge that the goals of these stakeholders are not entirely resonant. Although everyone benefits from a positive clinical trial and more effective treatment, the sponsor company's primary mission is to conduct a trial sufficient to meet regulatory requirements for approval and marketing of a therapy. Moreover, both a longer duration of administration and a higher drug cost translate to greater benefits to a company. In contrast, oncology professionals should seek a trial designed to deliver the maximal true clinical benefit to their patients relative to current best practice while ideally also being mindful of societal costs and hoping to minimize the duration and practical burdens of treatment for patients. These goals may well represent a higher standard than the threshold for regulatory approval. Meanwhile, patients understandably seek any new alternative offering a plausible improvement in efficacy and/or tolerability and have variable exposure to the costs of treatments.

The oncology community should also hope to maximize equity and treatment opportunities for patients with cancer globally, especially those participating in clinical research. We should hope to ensure that trials enforce global standards that elevate substandard staging and medical care practices rather than perpetuate and tacitly exonerate them. Variable practices of lower standards reduce the comparability of the control arm to contemporary optimal practice, compromising the scientific integrity of a trial. Unfortunately, suboptimal care in the control arm confers a fortuitous even if unintentional benefit for sponsor companies by amplifying differences between the control and experimental arms of the study. It also creates a dynamic in which clinical benefit predicated on poor access among those who cannot afford optimal treatment presents the mechanism to support greater access preferentially among those who are *already* beneficiaries of superior treatment options, amplifying disparities in cancer care.

## Fast Followers and Price Competition

If the high cost of a targeted therapy like osimertinib is prohibitive for many health care systems around the world, one potential path by which they may circumvent this is to develop and ultimately approve highly comparable agents that may have been developed specifically as low-cost, “me-too” alternatives. Here, the first-generation EGFR TKI, icotinib, provides a template for successful introduction of a clinically comparable alternative to other far more costly first-generation EGFR TKIs, gefitinib and erlotinib.^8^ The China-developed third-generation EGFR TKI, aumolertinib, has demonstrated superior PFS results compared with gefitinib in the AENEAS trial for first-line treatment of *EGFRm+* NSCLC^9^ that strongly echoes the findings of osimertinib in FLAURA.^5^ This agent offers the promise of a far less expensive but remarkably similar agent as osimertinib in some parts of the world, at least in advanced *EGFRm+* NSCLC, although there are no data anticipated for it or another late-generation EGFR TKI in the adjuvant setting in the foreseeable future.

Patients in some countries without feasible access to osimertinib may potentially be able to self-purchase off-label aumolertinib to use in the adjuvant setting, but we cannot presume with complete confidence that the same results seen in ADAURA would be achieved with substituted agents that have not undergone testing in analogous clinical trials. Duplicative trials such as AENEAS may ultimately be performed to confirm comparable utility for a less expensive agent such as aumolertinib as a financially feasible alternative to osimertinib. Nevertheless, we should question the equity of a global clinical trial framework that requires the redundancy and arguable waste of additional trials while incurring years-long time delays just to provide two-tiered access to therapy to a population of patients with cancer in low- and middle-income countries (LMICs) on which the global trial community depends to make the prohibitively costly therapies available earlier to patients in more resourced health care systems.

## Envisioning an Optimized ADAURA

Although we feel DFS was not the optimal primary end point for adjuvant therapy with an EGFR TKI even at the time the trial was conceived, OS became increasingly important once osimertinib became the preferred standard of care for patients with relapsed/metastatic *EGFRm+* NSCLC. In this setting, *mandating* crossover to osimertinib for patients in the control arm at the time of relapse would have simultaneously reflected practice patterns in the health care systems in which adjuvant osimertinib is to be marketed and provided the most ethical strategy to address disparities in global access to optimal care.

Moving forward, we would also strongly recommend that eligibility requirements for global trials maintain standards for optimal staging as per National Comprehensive Cancer Network guidelines,^10^ rather than sanctioning a variable and lower standard that omits positron emission tomography and/or brain magnetic resonance imaging scans when they would otherwise be indicated; this includes covering the costs of optimal staging if needed. Enrolling patients who have undergone substandard staging introduces a problematic bias against the control arm of an adjuvant therapy trial if it includes patients who have occult advanced disease that should have been detected by meticulous staging.

We submit that oncologists and patients should expect that phase III trials are conducted in a way that aspires to more than the lowest threshold of what a lenient regulatory authority will accept. Rather, the oncology community should seek that key trials enroll appropriately staged patients and ensure they receive access to the best treatments as defined by the contemporary practice in health care systems in which the experimental therapy would be marketed.

We work within a system of global trials in which cheaper, fast follower, or “me-too” drugs may be tested in trials that replicate findings of pivotal trials conducted merely to provide a financially feasible alternative for patient populations who do not have access to prohibitively costly agents like osimertinib. This creates a separate but equal distribution that requires patients in LMICs to complete redundant trials with fast follower drugs just to have access to the same benefits of patients in better resourced countries, typically after a delay of several years for the additional redundant work. We should acknowledge that our current processes that perpetuate geographic disparities in oncology are able to be refined in ways that can remove the confounding variable of access from timing of treatment while also providing more equitable care that ensures delivery of optimal standard-of-care treatment to patients who participate in cancer research.
